# Mapping the EQ-5D index by UPDRS and PDQ-8 in patients with Parkinson’s disease

**DOI:** 10.1186/1477-7525-11-35

**Published:** 2013-03-08

**Authors:** Judith Dams, Jens Klotsche, Bernhard Bornschein, Jens P Reese, Monika Balzer-Geldsetzer, Yaroslav Winter, Anette Schrag, Andrew Siderowf, Wolfgang H Oertel, Günther Deuschl, Uwe Siebert, Richard Dodel

**Affiliations:** 1 Department of Neurology, Philipps-University, Marburg, Germany; 2 Epidemiologic Unit, Leibniz-Institute, German Rheumatism Research Centre, Berlin, Germany; 3 Institute of Public Health, Medical Decision Making and Health Technology Assessment, Department of Public Health and Health Technology Assessment, UMIT - University for Health Sciences, Medical Informatics and Technology, Hall i.T., Austria; 4 Department of Clinical Neurosciences, Royal Free & University College Medical School, London, UK; 5 Avid Radiopharmaceuticals, Philadelphia, PA, USA; 6 Department of Neurology, Christian-Albrechts-University, Kiel, Germany; 7 Division of Public Health Decision Modelling, Health Technology Assessment and Health Economics, ONCOTYROL Center for Personalized Cancer Medicine, Innsbruck, Austria; 8 Institute for Technology Assessment and Department of Radiology, Massachusetts General Hospital, Harvard Medical School, Boston, MA, USA; 9 Department of Health Policy and Management, Harvard School of Public Health, Boston, MA, USA

**Keywords:** Parkinson’s disease, Quality of life, EuroQoL/EQ-5D, UPDRS, PDQ-8, Prediction

## Abstract

**Background:**

Clinical studies employ the Unified Parkinson’s Disease Rating Scale (UPDRS) to measure the severity of Parkinson’s disease. Evaluations often fail to consider the health-related quality of life (HrQoL) or apply disease-specific instruments. Health-economic studies normally use estimates of utilities to calculate quality-adjusted life years. We aimed to develop an estimation algorithm for EuroQol- 5 dimensions (EQ-5D)-based utilities from the clinical UPDRS or disease-specific HrQoL data in the absence of original utilities estimates.

**Methods:**

Linear and fractional polynomial regression analyses were performed with data from a study of Parkinson’s disease patients (n=138) to predict the EQ-5D index values from UPDRS and Parkinson’s disease questionnaire eight dimensions (PDQ-8) data. German and European weights were used to calculate the EQ-5D index. The models were compared by R^2^, the root mean square error (RMS), the Bayesian information criterion, and Pregibon’s link test. Three independent data sets validated the models.

**Results:**

The regression analyses resulted in a single best prediction model (R^2^: 0.713 and 0.684, RMS: 0.139 and 13.78 for indices with German and European weights, respectively) consisting of UPDRS subscores II, III, IVa-c as predictors. When the PDQ-8 items were utilised as independent variables, the model resulted in an R^2^ of 0.60 and 0.67. The independent data confirmed the prediction models.

**Conclusion:**

The best results were obtained from a model consisting of UPDRS subscores II, III, IVa-c. Although a good model fit was observed, primary EQ-5D data are always preferable. Further validation of the prediction algorithm within large, independent studies is necessary prior to its generalised use.

## Background

In recent years, increased measurement of health-related quality of life (HrQoL) has expanded to evaluate chronic disorders and analyse cost-effectiveness in particular. Different instruments were developed to assess the HrQoL. Types of HrQoL instruments include profile-based instruments that depend on the aggregation of several outcome values (e.g., Parkinson’s disease questionnaire eight dimensions (PDQ-8) [1]) and index instruments with a single index value to represent the HrQoL (e.g., EuroQol – 5 dimensions (EQ-5D) [2]). Disease-specific (e.g., PDQ-8 [1]) and generic instruments (e.g., EQ-5D [2]) are also available.

The guidelines for health-economic evaluations call for the implementation of quality of life as a patient-relevant outcome and the use of utility-based patient preferences [3-5]. However, utility-based instruments are not routinely applied, even in recent clinical trials. Clinical scales are regularly used, and study designs frequently include disease-specific HrQoL or profile instruments.

Cost-utility studies require HrQoL data, and clinical effectiveness parameters. We aimed to develop a mapping algorithm based on Unified Parkinson's Disease Rating Scale (UPDRS) and PDQ-8 data in cases when utilities are needed but not assessed in the field of Parkinson’s disease.

## Methods

### Clinical evaluation

The data were collected from a study population of patients (n=138) with idiopathic Parkinson’s disease following recruitment at several study centres in Hessia, Germany. A detailed description of the patients and scales applied was previously published [6]. The severity of Parkinson’s disease was assessed with the UPDRS [7]. Our analysis relied on subsets to calculate several scores, including the summed scores of parts II-IV. The latter data were also divided into subscores for dyskinesias (IVa), motor fluctuations (IVb), and other complications (IVc).

The HrQoL was evaluated with the generic EQ-5D and the disease-specific, profile-based HrQoL Parkinson’s disease questionnaire in its short version (PDQ-8) [1]. The health states identified by the EQ-5D were converted into EQ-5D indices employing weights from the German population valued with the time trade-off approach (hereafter referred to as the EQ-5D German_index_) ranging between 0 and 1 [8], and weights from a pooled European population valued by a visual analogue technique ranging from 0 to 100 (EQ-5D European_index_) [9].

We validated our results with three independent datasets: (1) our own unpublished data, (2) data from Siderowf et al. [10], and (3) data from Schrag et al. [11]. Siderowf et al. reported data for the UPDRS II and III, the PDQ-8, and the EQ-5D. The data from Schrag et al. consisted of the EQ-5D_index_, PDQ-8, and all UPDRS subscores. Our own data included the EQ-5D_index_ and the UPDRS II and III. We predicted the EQ-5D values with these independent data sets and calculated R^2^ resulting from the predicted and observed values.

The study protocol for our own data and data from Spottke et al. [6] was approved by the local ethics committee and all patients gave informed consent. Schrag et al. [11] obtained ethics approval from the National Hospital for Neurology and Neurosurgery and the Institute of Neurology Joint Medical Ethics Committee. The study provided by Siderwof et al. [10] was reviewed by the Research Review Committee of Pennsylvania Hospital, and informed consent was obtained from all subjects prior to administration of study instruments.

### Statistical analysis

A correlation analysis was calculated by a two-sided Spearman’s rank correlation test to determine any linear relationship between the predictor and the dependent variable. A multiple linear regression analysis was applied to develop a prediction rule for EQ-5D (i.e., German_index_ and European_index_) from the UPDRS and PDQ-8 variables. The interaction terms and squares of the variables were considered including PDQ-8- and UPDRS-subscores. Following the algorithm established by Cheung et al. [12], we built quadratic terms of these scales to consider non-linear relationships. We conducted a fractional polynomial regression analysis [13] to provide an alternative analytical approach to model the non-linear relationships between the outcomes and predictors. We investigated a logarithmic relationship and a relationship up to the third degree between the EQ-5D and the independent variables. The relationship between EQ-5D and predictor variables was nonparametrically estimated by a local polynomial smoothing of a general additive regression without making a functional assumption about the relationship. This approach serves as a graphical check of the parametric model fit to the data. In a second analysis, each EQ-5D dimension item was predicted, and the EQ-5D_index_ values were subsequently calculated. Several items of the UPDRS II-IV cover similar aspects as some EQ-5D items (e.g. activities of daily living/ self care by the UPDRS II or mobility, and pain by the UPDRS III). To investigate the relevance on the overall association between the UPDRS II-IV and the EQ-5D, we repeated our analyses after the elimination of UPDRS items 9, 10, 11, 12, 13, 14, 15, 17, 22, 29, 30, and 31 from the recalculated UPDRS II-IV scores.

Four basic regression models were built as follows:

$$\begin{array}{l} M1“\mathrm{UPDRS}\;\mathrm{II}-\mathrm{III}”:\\ \mathrm{EQ}-5D=\mathrm{UPDRS}\;\mathrm{II}+\mathrm{UPDRS}\;\mathrm{III}\\ M2“\mathrm{UPDRS}\;\mathrm{II}-\mathrm{IV}”:\\ \mathrm{EQ}-5D=\mathrm{UPDRS}\;\mathrm{II}+\mathrm{UPDRS}\;\mathrm{III}+\mathrm{UPDRS}\;\mathrm{IV}\\ M3“\mathrm{UPDRS}\;\mathrm{II}-\mathrm{IVa}-c”:\\ \mathrm{EQ}-5D=\mathrm{UPDRS}\;\mathrm{II}+\mathrm{UPDRS}\;\mathrm{III}+\mathrm{UPDRS}\;\mathrm{IVa}+\\ \mathrm{UPDRS}\;\mathrm{IVb}+\mathrm{UPDRS}\;\mathrm{IVc}\\ M4“\mathrm{PDQ}-8”:\\ \mathrm{EQ}-5D=\mathrm{PDQ}1+\mathrm{PDQ}2+\mathrm{PDQ}3+\mathrm{PDQ}4+\mathrm{PDQ}5+\\ \mathrm{PDQ}6+\mathrm{PDQ}7+\mathrm{PDQ}8 \end{array}$$

The models were constructed applying backward selection. For the model validation R^2^ and root mean square error (RMS) were calculated. To be consistent with other published work [12,14,15], we considered values for R^2^ ≥0.3 as acceptable and R^2^ values ≥0.5 as good predictions.

The alternative model fit was evaluated with the Pregibon link test [16] and the Bayesian information criterion (BIC). The model specification error was tested by the Pregibon link test to check the linearity of the EQ-5D on its prediction scale. The alternative model selection was assessed by the BIC. We graphically conducted a comparison of the linear regression analysis and factional polynomials against the local polynomial smoothing.

All analyses were calculated with the statistical packages STATA and R (Stata 12, StataCorp LP, Texas USA; R-2.15.1 Comprehensive R Archive Network, Institute for Mathematics, TU Vienna, Austria).

## Results

Seventeen patients were excluded because of missing data. We therefore evaluated a total of 121 patients. The mean patient age was 67.1 years (SD 9.1) and 66.1% were males. Approximately 2/3 of the population was classified into Hoehn&Yahr (HY) stage II, III or IV, with 6.6% in stage I and 6.6% in stage V. No differences in age and sex were observed between included and excluded cases but excluded cases had higher HY stages, with nearly 3/4 of these cases being in HY stages IV or V.

The correlation analysis demonstrated that the EQ-5D German_index_ and the EQ-5D European_index_ were associated for some variables: PDQ1, PDQ2, UPDRS II, UPDRS III with the EQ-5D German_index_, and PDQ1, PDQ2, PDQ7, UPDRS II, UPDRS III with the EQ-5D European_index_ (all r_s_ >0.6 and p <0.05).

On average, 50.0% (n=9) of the models analysed in “UPDRS II-III”, 42.6% (n=23) in “UPDRS II-IV”, 24.3% (n=118) in “UPDRS II-IVa-c” and 1.5% (n=197) in “PDQ-8” solely consisted of coefficients with a significant *p*-value (p <0.05). We will refer to these models as “significant models”.

The equations for best data fit of the EQ-5D German_index_ were represented by

$$\begin{array}{l} M1“\mathrm{UPDRS}\;\mathrm{II}-\mathrm{III}”:\\ \mathrm{EQ}-5D=0.9042-0.0001*\mathrm{UPDRS}\;\mathrm{II}I^{2}\\ M2“\mathrm{UPDRS}\;\mathrm{II-IV}”:\\ \mathrm{EQ}-5D=0.9275-0{.0001*\mathrm{UPDRS}\;\mathrm{III}}^{2}-0.0134\\ {}*\mathrm{UPDRS}\;\mathrm{IV}\\ M3“\mathrm{UPDRS}\;\mathrm{II}-\mathrm{IVa}-c”:\\ \mathrm{EQ}-5D=0.9628-0.0001*\mathrm{UPDRS}\;\mathrm{II}I^{2}+0.0031\\ {}*\mathrm{UPDRS}\;\mathrm{IV}a^{2}-0.0052*{\mathrm{UPDRS}\;\mathrm{IVb}}^{2}-00.0448\\ {*\mathrm{UPDRS}\;\mathrm{IVc}}^{2}\\ M4“\mathrm{PDQ}-8”:\\ \mathrm{EQ}-5D=0.9298-0.00004*\mathrm{PDQ}1^{2}-0.00002\\ {}*\mathrm{PDQ}2^{2}-0.00004*\mathrm{PDQ}8^{2} \end{array}$$

For the EQ-5D European_index_, we determined the following:

$$\begin{array}{l} M1“\mathrm{UPDRS}\;\mathrm{II}-\mathrm{III}”:\\ \mathrm{EQ}-5D=79.272-0.775*\mathrm{UPDRS}\;\mathrm{II}-0.008\\ {}*\mathrm{UPDRS}\;\mathrm{II}I^{2}\\ M2“\mathrm{UPDRS}\;\mathrm{II}-\mathrm{IV}”:\\ \mathrm{EQ}-5D=76.850-0.010*\mathrm{UPDRS}\;\mathrm{II}I^{2}-1.520\\ {}*\mathrm{UPDRS}\;\mathrm{IV}\\ M3“\mathrm{UPDRS}\;\mathrm{II}-\mathrm{IVa}-c”:\\ \mathrm{EQ}-5D=80.054-0.010*\mathrm{UPDRS}\;\mathrm{II}I^{2}+0.242*\\ \mathrm{UPDRS}\;\mathrm{IV}a^{2}-2.236*\mathrm{UPDRS}\;\mathrm{IVb}-3.919*\\ \mathrm{UPDRS}\;\mathrm{IV}c^{2}\\ M4“\mathrm{PDQ}-8”:\\ \mathrm{EQ}-5D=81.960.-0.380*\mathrm{PDQ}1-0.003*\mathrm{PDQ}2^{2}-\\ 0.003*\mathrm{PDQ}8^{2} \end{array}$$

The models were compared for the best data fit with maximum R^2^ values, and minimum RMS values. The model “UPDRS II-IVa-c” showed the best fit for both the EQ-5D German_index_ and the EQ-5D European_index_ (R^2^ = 0.712 and 0.684, respectively) (Table 1). The same model also showed the smallest RMS values (0.14 and 13.38, respectively). The R^2^ and RMS values for all other models for the EQ-5D were in the ranges of 0.538-0.603 (R^2^) and 0.16-0.17 (RMS) for the German_index_ and 0.561-0.666 (R^2^) and 13.75 to 15.78 (RMS) the EQ-5D European_index_ (Table 1). The elimination of similar items from the UPDRS II-IV resulted in R^2^ values of 0.684 for the German_index_ and 0.682 for the EQ-5D European_index_.

**Table 1:** Results from regression analysis

	**EQ-5D German**_**index**_								**EQ-5D European**_**index**_							
	**M1**		**M2**		**M3**		**M4**		**M1**			**M2**		**M3**		**M4**	
Intercept	0.904154***		0.927487***		0.962811***		0.9298***		79.272395***			76.85026***		80.05367***		81.959757***	
UPDRS II									−0.775300 * ^1^								
UPDRS III	−0.000129*** ^2^		−0.000111*** ^2^		−0.000106*** ^2^				−0.008005*** ^2^			−0.01006*** ^2^		−0.01022*** ^2^			
UPDRS IV			−0.013411** ^1^									−1.52015*** ^1^					
UPDRS IVa					0.003055** ^2^									0.24170* ^2^			
UPDRS IVb					−0.005198*** ^2^									−2.23550** ^1^		,	
UPDRS IVc					−0.044820*** ^2^									−3.91892*** ^2^			
PDQ1							−0.000043*** ^2^									−0.379520*** ^1^	
PDQ2							−0.000023* ^2^									−0.002511*** ^2^	
PDQ3																	
PDQ4																	
PDQ5																	
PDQ6																	
PDQ7																	
PDQ8							−0.000035*** ^2^									−0.003005*** ^2^	
**R**^**2**^	**0.5377**		**0.5685**		**0.7117**		**0.6034**		**0.5607**			**0.5850**		**0.6840**		**0.6662**	
**RMS**	**0.1743**		**0.1691**		**0.1394**		**0.1628**		**15.7759**			**15.3327**		**13.3798**		**13.7519**	
**Link test**	**0.068**	**n.s.**	**0.053**	**n.s.**	**0.33**	**n.s.**	**0.053**	**n.s**	**0.772**	**n.s.**	**0.025**	**n.s.**	**0.999**	**n.s.**	**0.025**	**n.s.**	

Statistics for the regression analysis including the model fit of models achieving a maximum R^2^ and minimum root mean square error (RMS);

* represent the level of significance (n.s.: not significant for p ≥0.05, *: p <0.05, **: p <0.01, ***: p <0.001); the numbers indicate the degree of terms included in the model for maximum R^2^ (^1^linear terms, ^2^quadratic terms); PDQx indicates the respective of the eight items of the PDQ-8 questionnaire.

The model structure and complexity was evaluated by the goodness of the link-test of Pregibon [16] and the BIC. The link test did not reject the hypothesis of model misspecification for all models constructed. This result indicates that the functional relationship was correctly specified for all significant predictors considered in the model. The smallest coefficients were observed for the “UPDRS II-IVa-c” model regardless of the European_index_ or German_index_ prediction (Table 1). This result was further supported by a small BIC for the M3 model.

**Figure 1:**
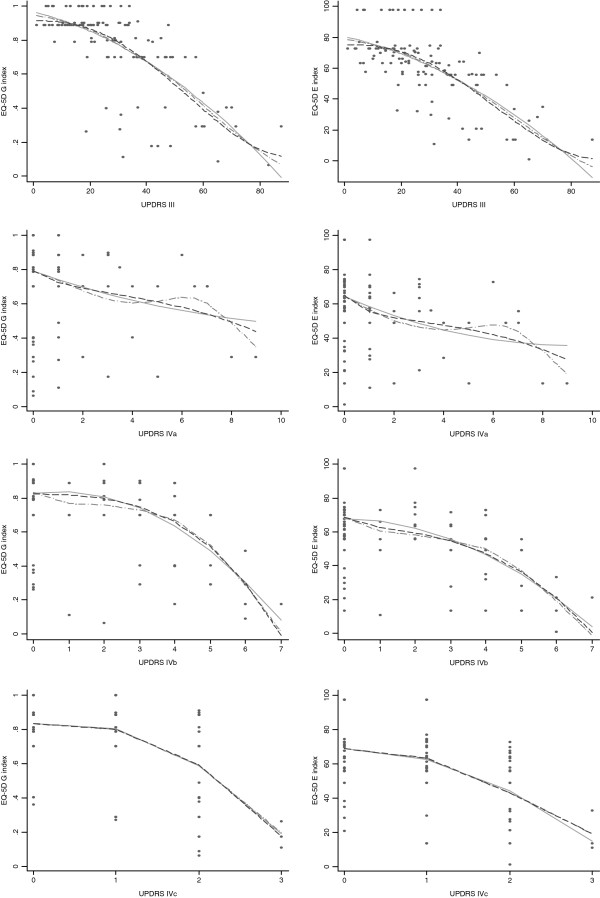
**Presentation of the EQ-5D data fit for the regression line (left panel: EQ-5D German**_**index**_**; right panel: EQ-5D European**_**index**_**): comparison of the fitted values estimated by alternative analytic approaches (dots: EQ-5D values; solid line: fitted regression line by ordinary linear regression; dash-dot line: fitted regression line by a generalised additive model; dashed line: fitted regression line by fractional polynomials). The UPDRS II was not presented because its categorical nature led to the accumulation of points by a small number of values.**

The fractional polynomial regression resulted in the same models with optimal R^2^. The original EQ-5D data and a graphical comparison of the estimated regression models (linear, fractional polynomial and general additive regression) are shown in Figure 1.

The regression analysis for the single EQ-5D questions 1–5 resulted in a R^2^ of 0.31 for the EQ-5D European_index_ and 0.26 for the EQ-5D German_index_ items.

The validation of our results with independent data from our own (M1 model), from Siderowf et al. [10] (M1 and M4 models) and Schrag et al. [11] (all models) showed R^2^ values ranging from 0.11 to 0.56 for the EQ-5D German_index_ and from 0.24 to 0.64 for the EQ-5D European_index_. These results confirm the results (except for the prediction of the EQ-5D German_index_ by the UPDRS II-III model) from our primary data showing robust results and indicating external validity.

## Discussion

We present an algorithm for the estimation of the EQ-5D from the UPDRS parts II-IV and the PDQ-8, both of which are standard clinical classification schemes that are widely used in the evaluation of Parkinson’s disease patients within clinical studies. Our prediction models based on the UPDRS explained more than 71% and 68% of the variation and used models having minimal RMS of 0.14 and 13.38 in the EQ-5D German_index_ and EQ-5D European_index_, respectively. The results were reproduced by our own independent data and data from Siderowf et al. [10] and Schrag et al. [11]. We note, however, that the application of empirical utility data is preferable if available. However, we address an approach to these issues when utility data are missing.

Our mapping algorithm for the UPDRS compared to the PDQ-8 explained slightly more of the appearing variance predicting the EQ-5D (PDQ-8: 60.3% and 66.6%; UPDRS: 71.2% and 68.4% for the EQ-5D German_index_ and the EQ-5D European_index_). This finding was supported by the RMS (PDQ-8: 0.16 and 13.75, UPDRS: 0.14 and 13.38 for the EQ-5D German_index_ and EQ-5D European_index_). This result is surprising because we expected the PDQ-8 by measuring Parkinson specific quality of life to have a greater conceptual resemblance to the EQ-5D. The fractional polynomial regression tested different types of models, and we concluded that the “PDQ-8” data have a poorer fit compared to “UPDRS II-IVa-c”. One possible explanation for this result is the different nature of the items in the two instruments; the EQ-5D has a stronger focus on the perceived impaired general health due to the physical illness, and the PDQ-8 considers more of the social and psychological consequences of Parkinson’s disease. The focus of the UPDRS on physical constraints makes this instrument more likely to have a relationship conceptually closer to the EQ-5D. Additional analyses showed that similar items in the UPDRS II-IV and the EQ-5D did not have a relevant impact on the association between the UPDRS and the EQ-5D, thus supporting our potential explanation. The link test and the BIC indicated that the model includes all important terms (see also Figure 1). Although we do not expect to find a relevant bias, we cannot completely rule out residual bias and model misspecification. The maximal R^2^ and minimal RMS represent the best fit of the data, but not necessarily the most logical relationship between the predictor and the independent variables investigated. The unexplained variance of approximately 30% may result from conceptual differences between the scales (e.g., in comorbidities such as depression) or differences in the evaluation technique (i.e., self- vs. professional-rating). Furthermore regression analysis does not consider pseudo-correlation or multi-collinearity.

We attempted to detect country-specific responses to the EQ-5D questionnaire with an analysis of the EQ-5D with German and European weights, but the marginal differences indicate the robustness of our models.

In contrast, when the model suggested by Cheung et al. [12] was applied to our data it failed to result in a satisfying model fit (R^2^ close to zero and RMS = 3651.5), suggesting that the model is inappropriate for our data. However, Cheung et al. calculated an Asian EQ-5D_index_, necessitating a careful comparison between our German data and Cheung’s et al. results. We therefore expanded our analysis beyond the work of Cheung et al. and analysed the quadratic, cubic and logarithmic relationships between the EQ-5D_index_ and PDQ-8 or UPDRS. However, non-linear effects did not contribute to the association in a relevant way.

Another recently published study [14] dealt with the prediction of EQ-5D dimensions from PDQ-39 items using sophisticated simulation-based methods. The authors showed a better prediction with their method compared to several regression analysis methods. This is consistent with our results for the prediction of EQ-5D items 1–5, which resulted in R^2^ of 0.26 for the EQ-5D German_index_ and 0.31 for the EQ-5D European_index_. However, the approach described by Borchani et al. is probably not easily applicable in the clinical setting.

## Conclusion

The EQ-5D_index_ values were best estimated with a model based on the UPDRS subscales II-IVa-c regardless of whether we applied German or European weights to calculate the EQ-5D. The data fit as measured by the maximum R^2^ and minimum RMS is best for these models. The prediction rule could be validated with several independent data sets, indicating the potential for general usefulness. However, the results from the application of the instrument in large and independent studies should be reported prior to general application.

## Abbreviations

BIC: Bayesian information criterion; EQ-5D: EuroQol – 5 dimensions; HrQoL: Health-related quality of life; HY: Hoehn & Yahr stage; PDQ-8: Parkinson’s disease questionnaire eight dimensions; RMS: Root mean square error; UPDRS: Unified Parkinson’s disease rating scale.

## Competing interests

This study was supported by a research grant from the German Federal Ministry of Education and Research/Parkinson Competence Network, 01GI9901/1 and the German Parkinson Association. Prof. Siebert’s work was in part supported by the COMET Center ONCOTYROL, which is funded by the Austrian Federal Ministries BMVIT/BMWFJ (via FFG) and the Tiroler Zukunftsstiftung/Standortagentur Tirol (SAT). Andrew Siderowf is an employee of Avid Radiopharmaceuticals, a wholly owned subsidiary of Eli Lilly and Co. No competing financial and non-financial interests exist which adversely affected the preparation of this manuscript.

## Authors’ contributions

JD was involved in the conception, organization and execution of the research project. She evaluated the design of statistical analysis and has written the first draft of this article. JK supported the statistical analysis in design and execution and reviewed the article critically. BB was involved in the execution of the research project and the reviewing of the article. JPR organized the research project and reviewed the article. MBG and YW reviewed the article. AS provided data for the analysis and reviewed the article. WHO reviewed the article. GD provided data for the analysis and reviewed the article. US was responsible for the execution of the research project, the design of statistical analysis and reviewed the manuscript. RD was involved in the conception, organization and execution of the research project. He supported the development of the statistical design and reviewed the article. All authors read and approved the final manuscript.
